# Personal

**Published:** 1894-05

**Authors:** 


### Personal.

Dr. J. B. S. Holmes, of Rome, Ga., has been elected president of the Tri-State Medical Association, that comprises the states of Tennessee, Alabama and Georgia. He has also been elected one of the vice-presidents of the Southern Surgical and Gynecological Association.

---

Dr. George Frederick Hulbert, of St. Louis, has removed his office and residence to 4270 Delmar avenue, south-east corner of 43d street, St. Louis, Mo. Hours: 10–12 a. m., 5–7 p. m. Telephone, 7202.

